# Supplementary material for: Foot-and-Mouth Disease Virus Capsid Protein VP1 Antagonizes Type I Interferon Signaling via Degradation of Histone Deacetylase 5
Source: Cells. 2024 Mar 19;13(6):539. doi: 10.3390/cells13060539 (PMC10969541; doi:10.3390/cells13060539)
Supplement: Supplementary file 1 [file cells-13-00539-s001.zip › cells-2847276-supplementary.pdf]

Supplementary Information

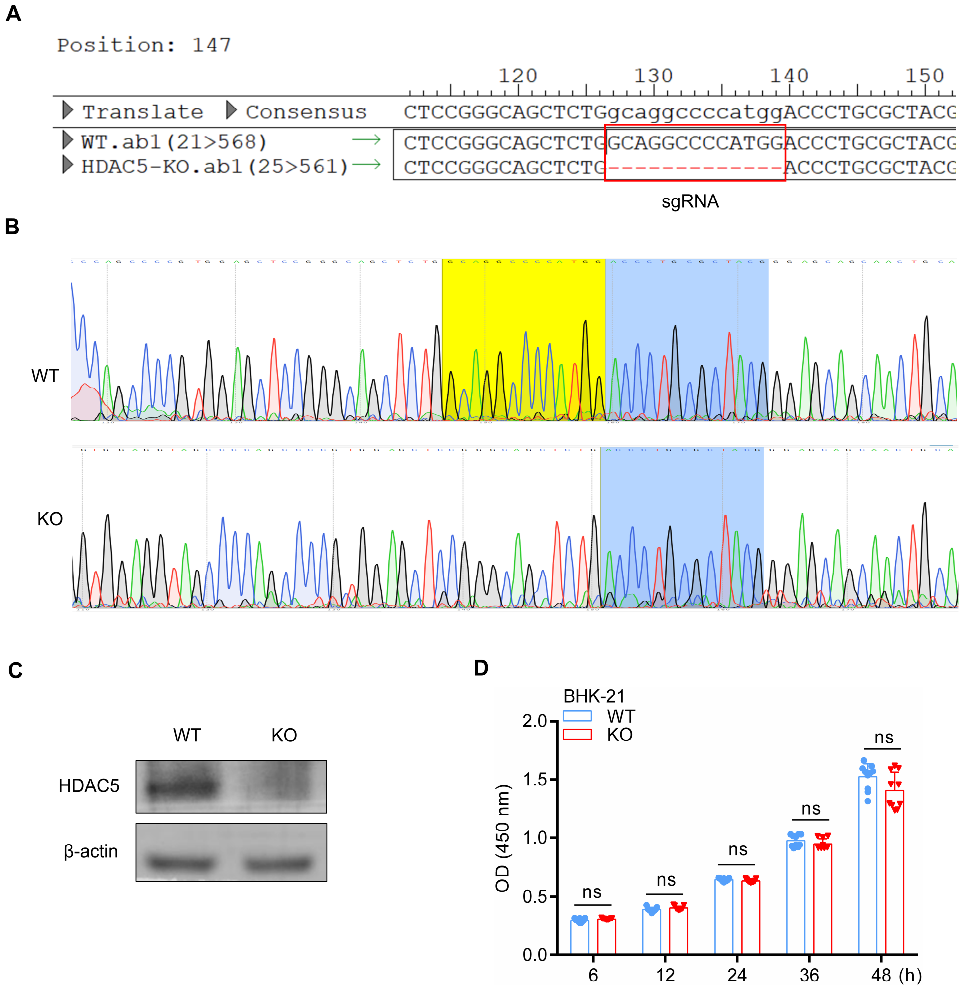

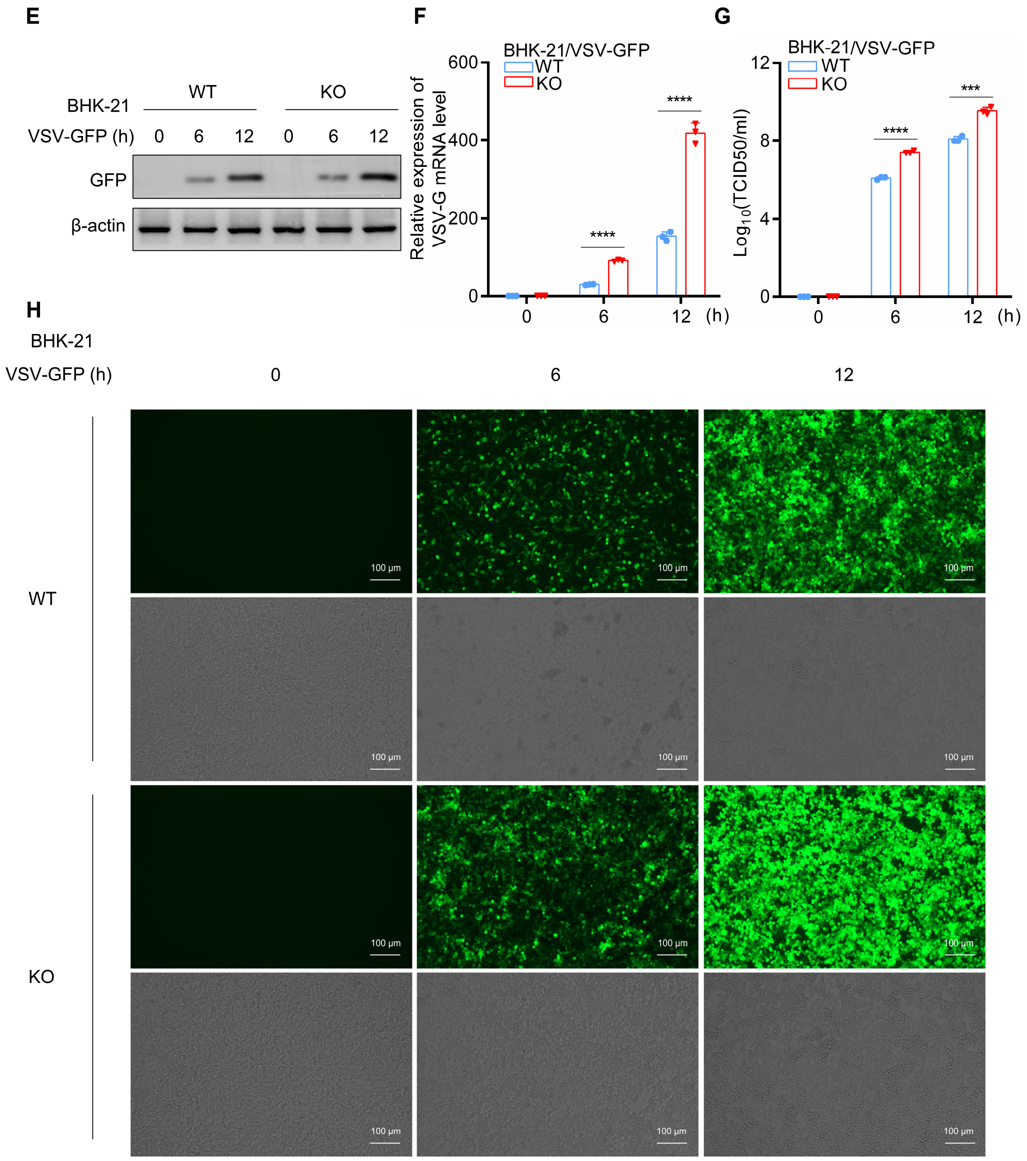

Figure S1. HDAC5 knockout promotes VSV replication. (**A**) The DNAMAN software was used to align the CDS sequences of WT and HDAC5-KO. The sgRNA sequence and mutations in the PAM motif are indicated by the red box. (**B**) Snapgene software was utilized to analysis the sequencing peaks of PCR products of WT and HDAC5-KO. (**C**) WT and HDAC5-KO BHK-21 cells were separately harvested. Western blotting was used to evaluate protein expression of HDAC5 and β-actin. (**D**) In 96-well plates, WT and HDAC5-KO BHK-21 cells were seeded. 10 μL of CCK8 reagent was used and cells were incubated for 60 minutes. Then, the cells were measured the absorbance at 450 nm. (**E-H**) WT and HDAC5-KO BHK-21 cells (1×10^6) were infected with VSV-GFP. Subsequently, cells and culture supernatants were separately harvested at the indicated time points. (**E**) Western blotting was used to evaluate β-actin and VSV-GFP protein expression. (**F**) RT-qPCR was applied to quantify the mRNA level of VSV-GFP. (**G**) $TCID_{50}$ was employed to test viral titers of VSV-GFP. (**H**) Fluorescence microscopy was employed to observe and photograph viral replication of VSV-GFP. Groups were compared by unpaired Student's *t*-test. ns, not significant, $p > 0.05$, p<0.01**, p<0.001***, p<0.0001****.

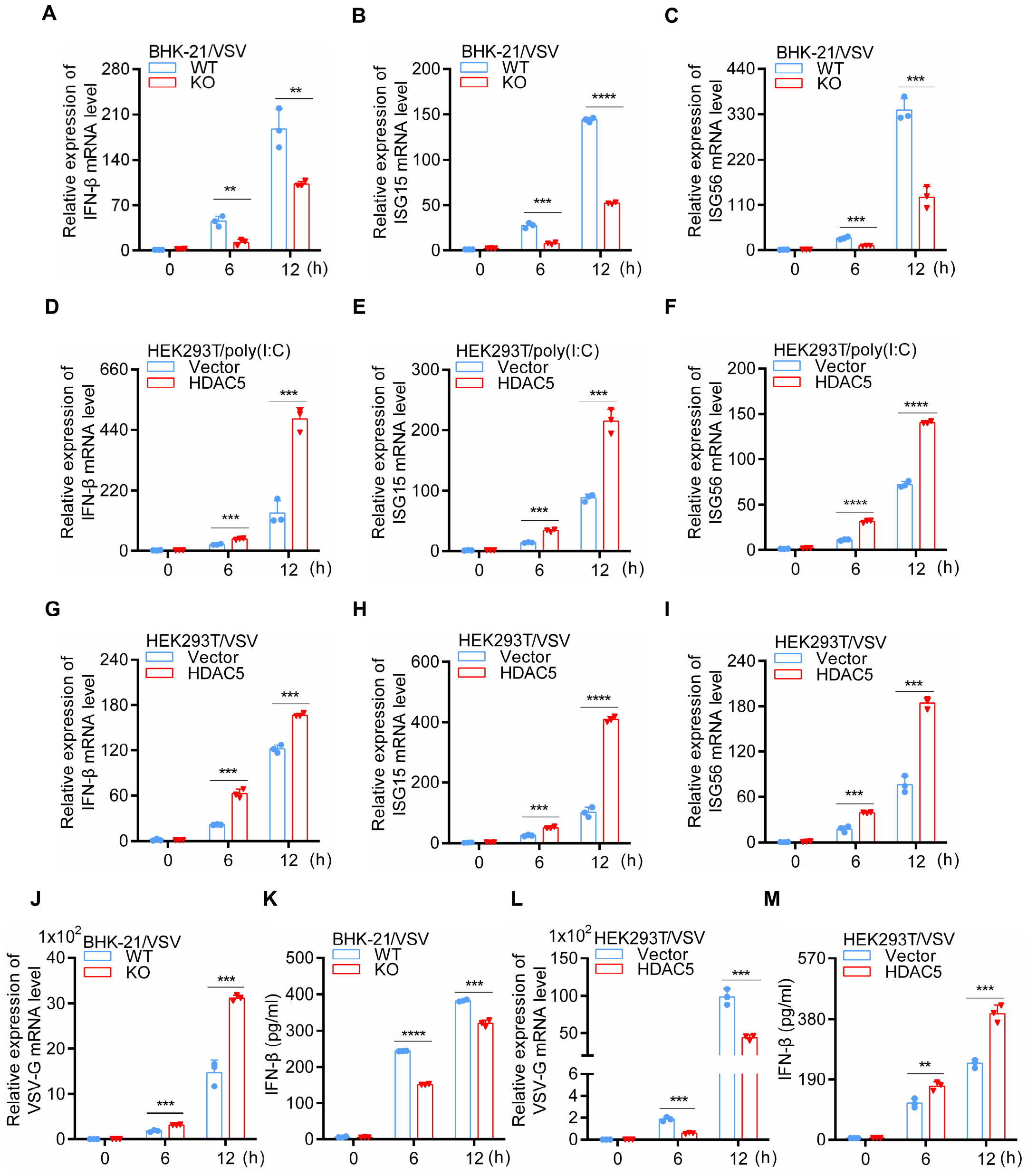

Figure S2. HDAC5 promotes poly(I:C)- and VSV-stimulated IFN-β signaling. (**A-C**) WT and HDAC5-KO BHK-21 cells (1×10^6) were infected with VSV. Subsequently, the cells were harvested separately at the indicated time points. RT-qPCR was applied to quantify the mRNA levels of IFN-β, ISG15, and ISG56. (**D-F**) HDAC5 or empty-vector plasmid (2 μg) was introduced into HEK293T cells (1×10^6) and incubated for 36 h, followed by poly(I:C) transfection. Subsequently, the cells were harvested separately at the indicated time points. RT-qPCR was applied to quantify the mRNA levels of IFN-β, ISG15, and ISG56. (**G-I**) HDAC5 or empty-vector plasmid (2 μg) was introduced into HEK293T cells (1×10^6) and incubated for 36 h, followed by VSV infection. Subsequently, the cells were harvested separately at the indicated time points. RT-qPCR was applied to quantify the mRNA levels of IFN-β, ISG15, and ISG56. (**J, L**) RT-qPCR was applied to quantify the mRNA levels of VSV-G (**J** as in **A-C**, **L** as in **G-I**). (**K, M**) Culture supernatants were separately harvested at the indicated time points, and ELISA was employed to detect IFN-β secretion (**K** as in **A-C**, **M** as in **G-I**). Groups were compared by unpaired Student's

t-test. ns, not significant, $p<0.01$**,$p<0.001$***, $p<0.0001$****.

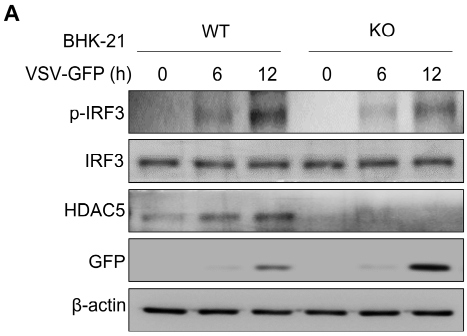

Figure S3. HDAC5 activates VSV-induced phosphorylation of IRF3. (**A**) WT and HDAC5-KO BHK-21 cells (1×10^6) were infected with VSV-GFP. Western blotting was used to evaluate protein expression of phosphorylated IRF3, total IRF3, HDAC5, VSV-GFP, and β-actin.
